# 2D Kinetic Analysis of TCR and CD8 Coreceptor for LCMV GP33 Epitopes

**DOI:** 10.3389/fimmu.2018.02348

**Published:** 2018-10-15

**Authors:** Elizabeth M. Kolawole, Rakieb Andargachew, Baoyu Liu, Jesica R. Jacobs, Brian D. Evavold

**Affiliations:** ^1^Department of Pathology, Microbiology and Immunology, University of Utah, Salt Lake City, UT, United States; ^2^Department of Microbiology and Immunology, Emory University, Atlanta, GA, United States

**Keywords:** TCR:pMHC, affinity, catch bond, GP33, LCMV, super agonist, immunodominance, CD8 T cell

## Abstract

The LCMV GP33 CD8 epitope has long been one of the most widely used antigens in viral immunology. Of note, almost all of the *in vitro* analyses of CD8 T cell responses to this epitope make use of an altered peptide ligand (APL) in which the cysteine from the original 9-mer peptide (KAVYNFATC) is substituted by a methionine at position 41 (KAVYNFATM). In addition, it is possible that the antigen processed during natural LCMV infection is an 11-mer peptide (KAVYNFATCGI) rather than the widely used 9-mer. Although previous affinity measurements using purified proteins for these antigen variants revealed minimal differences, we applied highly sensitive two dimensional (2D) biophysical based techniques to further dissect TCR interaction with these closely related GP33 variants. The kinetic analyses of affinity provided by the 2D micropipette adhesion frequency assay (2D-MP) and bond lifetime under force analyzed using a biomembrane force probe (BFP) revealed significant differences between 41M, 41C and the 11-mer 41CGI antigen. We found a hierarchy in 2D affinity as 41M peptide displayed augmented TCR 2D affinity compared to 41C and 41CGI. These differences were also maintained in the presence of CD8 coreceptor and when analysis of total TCR:pMHC and CD8:pMHC bonds were considered. Moreover, the three ligands displayed dramatic differences in the bond lifetimes generated under force, in particular the 41CGI variant with the lowest 2D affinity demonstrated a 15-fold synergistic contribution of the CD8 coreceptor to overall bond lifetime. Our analyses emphasize the sensitivity of single cell and single bond 2D kinetic measurements in distinguishing between related agonist peptides.

## Introduction

CD8+ cytotoxic T lymphocytes (CTLs), which recognize peptides presented by major histocompatibility complex (MHC) class I molecules, are critical for the antigen specific clearance of viral infections (1, 2). All CTL responses are dependent on recognition of the viral peptide, followed by sufficient triggering of the TCR to induce a cascade of signaling events (3). Thus, the initial interactions of TCR with pMHC are central to the recruitment of the adaptive immune arm of T cell mediated immunity. Consequently, the affinity of TCR for pMHC and other proximal parameters such as macromolecular orientation (4, 5), mechanosensing (6, 7), stability of the TCR:pMHC complex (8, 9), bond lifetime under force (9–11) and segregation of phosphatases from the T cell:APC synapse (9, 12–15) are critical in determining the efficiency of T cell differentiation and effector functions.

A major question that persists is how T cells can simultaneously possess a high level of specificity coupled with extreme sensitivity for as few as a single pMHC molecule (16, 17). This question has led to a number of models and technologies seeking to explain how TCR recognition of various pMHC complexes leads to such functionally different outcomes, giving rise to agonists and antagonist classifications (18–20). Affinity and T cell kinetics of TCR:pMHC can be acquired by surface plasmon resonance (SPR) which measures the receptor:ligand interaction in three dimensions (3D) (21), but this method lacks the sensitivity to measure the entire gamut of pMHC ligands, especially the lower affinity interactions. In contrast to using purified proteins for 3D measurements where the receptor:ligand interaction is isolated from cells, 2D based measures incorporate the proteins into the cellular membrane and are assessed at the cell-cell junctions, providing an added biological component to the interactions. More importantly, the 2D measurements possess increased levels of sensitivity to measure lower affinity and shorter bond lifetime interactions (6, 22, 23).

Lymphocytic choriomeningitis virus (LCMV) is one of the best characterized viral model systems in mice (24–26). LCMV has been key in impacting our overall understanding of T cell immunology responsible for many seminal findings including, but not limited to: peptide:MHC restriction, the kinetics of primary and memory T cell responses, viral epitope escape, T cell exhaustion and the role of PD-1, (27–30). An important feature is the existence of several well characterized strains conferring either a chronic (Clone 13 strain) or acute (Armstrong strain) viral infection (25, 31). During the response to LCMV infection, the majority of CD8+ CTLs are directed against three viral immunodominant H-2D^b^ MHC class I epitopes (in C57BL/6 mice); GP_33−41_, GP_276−286_, and NP_396−404_ (32, 33). Both the 11-mer GP_276−286_ and the 9-mer NP_396−404_ have a single optimal sequence. However, GP33 has been analyzed using several epitopes: the 9-mer 41M, GP_33−41_ (KAVYNFATC) (41C) and the 11 amino acid long GP_33−43_ (KAVYNFATCGI) (41CGI) (8).

The altered peptide ligand (APL) 9-mer 41M, was created by introducing a single methionine at the carboxy terminal end replacing the cysteine at position 41, (27, 34, 35). The terminal cysteine has been shown to form peptide dimers and has decreased stability in the MHC (27, 34–36). These data showed an increase in MHC class I binding from 41C to 41M (37). Therefore, most analyses of the LCMV GP33 D^b^ response have been analyzed with the more stable APL. Because of these changes in the peptide antigen used for the LCMV response, we sought to determine how the three immunodominant GP33 epitopes alter the 2D kinetics of TCR recognition of the respective antigens and the CD8 T cell functional response. In addition to the TCR:pMHC interaction we wanted to investigate the contribution of CD8 coreceptor to the TCR signaling. When blocked or in the absence of CD8 coreceptor, CD8+ T cells require longer and more substantial TCR engagement (38, 39). It has been suggested that CD8 binding helps weak ligands with low TCR:pMHC affinities (40). Furthermore, it has been demonstrated that CD8 binds sequentially following TCR:pMHC engagement (41). Here, we demonstrate the distinct hierarchy of 2D kinetics for the three viral variants that has been absent in 3D kinetic analyses. In addition, we found the contribution of CD8 coreceptor to bond lifetime under force strikingly increases with the weaker 41CGI variant. Our work demonstrates that 2D analysis can sensitively distinguish differences in closely related agonist peptides.

## Materials and methods

### Mice

C57BL/6 (B6) mice were purchased from the National Cancer Institute (NCI) and Charles River. P14 (Thy 1.2) TCR transgenic mice were housed and bred at the University of Utah. All animal experiments were conducted with the approval of the Institutional Animal Care and Use Committee at the University of Utah. C57BL6 Thy 1.1 congenic mice used as hosts for adoptive transfers were purchased from NCI. All mice were between 8 and 12 weeks of age.

### Virus

Lymphocytic choriomeningitis virus (LCMV) Armstrong were kindly provided by Dr. Matthew Williams (University of Utah) and was made as described (42).

### Peptides

GP33-41M (KAVYNFATM), GP33-41C (KAVYNFATC) and cognate GP33−43 (KAVYNFATCGI) peptides were synthesized at the University of Utah on the Prelude X peptide synthesizer (Protein Technologies).

### Adoptive cell transfer

Naïve CD8+ T cells were isolated from spleens of P14 (Thy 1.2) transgenic mice using MACS CD8+ T cell magnetic separation kit (Miltenyi Biotec) and intravenously transferred to congenic C57BL/6 (Thy1.1) hosts. Mice were infected with 2 × 10^5^ PFU of Armstrong by intraperitoneal (i.p.) injection 24 h after adoptive cell transfer.

## LCMV infections

8–12 weeks old C57BL/6 mice were injected i.p. with 2 × 10^5^pfu Armstrong and sacrificed at 8 days post infection. Spleens were harvested and stimulated with either KAVYNFATM (41M), KAVYNFATC (41C) or KAVYNFATCGI (41CGI) for 1 h and then intracellular cytokine staining was performed.

## 2D micropipette adhesion frequency assay (2D-MP)

The relative 2D affinity of naïve P14 H2D^b^ GP33 specific CD8+ T cells was measured using the previously characterized 2D-MP (6, 43–47). In brief, RBC's were coated with Biotin-LC-NHS (BioVision) followed by streptavidin (Thermo Fisher Scientific) and either biotinylated pMHC GP_33−41M_ (KAVYNFATM), GP_33−41C_ (KAVYNFATC) or GP_33−43_ (KAVYNFATCGI) monomers. For relative 2D affinity measurements of TCR:pMHC interaction, monomers with the D^b^ D227K mutation were used abrogating CD8 binding to MHC or without the D^b^ D227K mutation measuring normalized adhesion bonds of the TCR:pMHC:CD8 trimolecular interaction. All monomers were obtained from the NIH Tetramer Core Facility. In both sets of experiments the adhesion frequency between a single T cell and a ligand coated RBC aspirated on opposing pipettes was observed using an inverted microscope. An electronically controlled piezoelectric actuator repeated a T cell contact and separation cycle with the pMHC coated RBC 50 times while keeping contact area (A_c_) and time (t) constant. Upon retraction of the T cell, adhesion (binding of TCR:pMHC) was observed as a distention of the RBC membrane, allowing for quantification of the adhesion frequency (Pa) at equilibrium. Surface pMHC (m_l_) and TCR (m_r_) densities were determined by flow cytometry using an anti-TCRβ PE antibody (H57-597; BD Biosciences) and an anti-H2D^b^ antibody (clone:28-14-8; eBioscience) both at saturating concentrations along with BD QuantiBRITE PE beads for standardization (BD Biosciences). The calculation of molecules per area was determined by dividing the number of TCR and pMHC per cell by the respective surface areas. The relative 2D affinities were calculated using the following equation: A_c_K_a_ = –ln [1-P_a_(1)]/m_r_m_l_. Normalized adhesion frequency was calculated using the equation [–ln(1-Pa(s))/m_l_ (pMHC)]. Geometric means of all measured single cell affinities and normalized adhesion bonds are reported ± SEM. The centerpiece of our micropipette system is an Olympus IX71 inverted microscope equipped with fluorescence and a 100X oil immersion phase contrast and suspended on a TMC CleanBench vibration isolation table. The micropipettes are mounted onto the stage by adapters made by Narishige and are controlled by both fine and coarse micromanipulators. One micropipette is attached to the piezoelectric actuator via a Physik Instrumente P-840.1 piezo amplifier control module that is controlled by LabVIEW software on the imaging workstation. Micropipettes are held by Narishige HI-7 injection holders affixed by the heads of Narishige UT-2 universal joints (with joint removed) on custom mounts. Aspiration pressure of the micropipettes is maintained by Kontes water columns on height adjustable Velmex Unislide height adjusters. Micropipettes are produced on a Sutter Instruments P-1,000 pipette puller and finished using a Narishige MF-900 microforge.

### Biomembrane force probe assay (BFP)

Bond lifetime measurements under force were captured using the biomembrane force probe Assay (BFP). Procedures for coupling pMHC to glass beads have been described previously (10, 48). In brief, RBCs were first biotinylated with EZ-link NHS-PEG-Biotin (Thermo Fisher Scientific) and then reacted to streptavidin. Borosilicate beads were first cleaned, silanized, and then reacted to streptavidin-maleimide (Sigma-Aldrich, St. Louis, MO). Streptavidin beads were then coated with biotinylated pMHC either GP_33−41M_ (KAVYNFATM), GP_33−41C_ (KAVYNFATC) or GP_33−43_ (KAVYNFATCGI) monomers and placed on the apex of an RBC that was aspirated onto a micropipette. This bead served as a force probe. The position of the edge of the bead was tracked by a high-resolution camera (1,600 frames/s) with < 3 nm displacement precision. The T cell of interest was brought into contact with the glass bead, then retracted a set distance and held by the computer-controlled piezoelectric actuator. The retraction and hold-phase generated a force on the TCR:pMHC bond, which can be altered based on the distance the T cell is retracted. The camera then recorded the time it took for the T cell to disengage the glass bead, which was visualized as the RBC retracted and the bead returned to its starting position. Repeated cycles (known as force-clamp cycles) can be carried out at a single force in order to generate an average bond lifetime between the TCR and pMHC complex. For an optimal response to antigen, the bond lifetime increases with increasing force before reaching a peak bond lifetime, which is typical of a catch bond physiology. By varying the force and measuring the bond lifetimes one can determine what type of bond occurred. Bond lifetimes were analyzed as described (47) using a customized package run by LabVIEW (National instruments) first described by Chen et al. (49). The BFP is built around a Carl Zeiss Axio Observer A1 inverted microscope with two Narishige three-axis hanging joystick oil micromanipulators that allow for precise control of the micropipettes. One micropipette is attached to a Physik Instrumente piezoelectric actuator with nanometer resolution that is controlled with software programed on LabVIEW. To aspirate cells onto the micropipettes, we have two Eppendorf CellTram Vario pressure pistons, an Eppendorf FemtoJet for ejection of the cells and an engineered hydrostatic force pressure system. For data capture and analysis, we have two Allied Vision Technology cameras, one high speed (1,600 fps) and one normal speed (32fps) allowing for general recording and viewing (normal) with simultaneous recording of Brownian motion to detect formation and lifetime of individual bonds (high speed). In addition, we have an Andor iXon EMCCD Camera with sensitivity to a single photon. This is all housed on a Newport Vibration isolation table in a vibration-free room (~100 sq ft). Twins screens on the computer allow for real time tracking of bond formation (LabVIEW left screen) and view of the micropipettes (right screen).

### Intracellular cytokine staining

Splenocytes isolated from infected mice were plated at 2 × 106 cells per well in a 96-well plate and cytokine production was tested in response to either 41M, 41C or 41CGI peptides. Tested concentrations ranged from 1 uM to.03 nM in various dilutions. Cells were incubated with peptide for 1 h at 37° Celsius in R10 media and washed before Brefeldin A was added (MP Biomedicals) and the cells incubated for another 4 h. R10 media was composed of RPMI 1640 (Mediatech), 10% heat inactivated FBS (Hyclone), 10 mM HEPES buffer (Mediatech), 2 mM L-glutamine (Mediatech), 50 μM 2-mercaptoethanol (2ME) (Sigma), and 100 μg/ml gentamicin (Mediatech). Additional samples were also cultured without peptide as a negative control and stimulation with a PMA (20 nM; Fisher Biotech) ionomycin (1 μM; Sigma) combination was used as a positive control. Cells were then washed and stained with surface antibodies in the dark for 30 min on ice in FACS staining buffer composed of phosphate buffered saline (PBS) (Mediatech), 0.1% bovine serum albumin (BSA) (Fisher Scientific), and 0.05% sodium azide (Sigma). Surface markers were stained with anti-CD90.2 FITC (53-2.1; Biolegend), anti-CD44 PerCP Cy5.5 (IM7; BD), anti-CD3 Brilliant Violet 605 (145.2C11; Biolegend) anti-CD4 Brilliant Violet 711 (RM4-5; Biolegend) and anti-CD8 Brilliant Violet 785 (53.6.7; Biolegend). Using Tonbo bioscience Fix/Perm kit, cells were fixed and permeabilized as per manufacturer's protocol. Intracellular antibody staining with anti-IFNγ APC-Cy7 (XMG1.2; BD), anti-TNFα PE-Cy7 (MP6-XT22; Biolegend), anti-IL-2 APC (JES6-5H4; BD), anti-Nur77 PE (12.14; ebioscience) and anti-IRF4 eFluor 450 (3E4;Invitrogen) antibodies was performed as per manufacturers' protocol in a permeabilization buffer for 30 min on ice. Cells were washed and kept on ice before being run on the LSRFortessa X-20 cell analyzer (Beckton Dickson). All flow cytometry data were analyzed using FlowJo software (Treestar).

### Statistics

Statistical significance of measured values was determined by Ordinary one-way ANOVA and Tukey's multiple comparison test using the Prism Software (GraphPad). Statistical significance indicated as ns = no significance, ^*^P > 0.05, ^**^P > 0.01, ^***^P > 0.001, and ^****^P > 0.0001.

## Results

### Truncation (41C) and mutation (41M) of position 41 of the immunodominant GP33 epitope (41CGI) result in augmented 2D affinity

**Figure 1:**
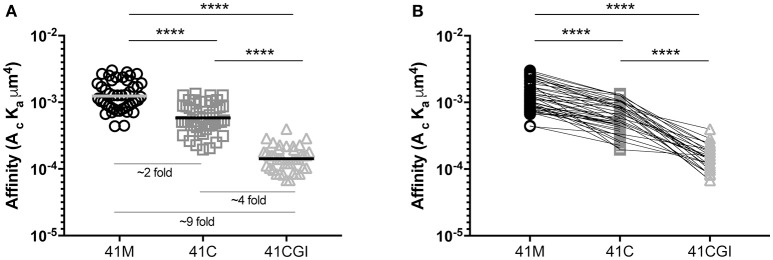
Truncation (41C) and mutation (41M) of position 41 of the naturally processed immunodominant GP33 epitope (41CGI) result in augmented 2D affinity. 2D affinity of naive CD8+ P14 splenocytes tested to either D^b^ GP_33−41M_, D^b^ GP_33−41C_ or D^b^ GP_33−41CGI_ monomers carrying the D^b^ D227K mutation (CD8-null). **(A)** shows the overall population mean affinity ± SEM while **(B)** shows the 2D affinity of a single P14 CD8+ T cell sequentially tested against each pMHC monomer (test sequence randomized from cell to cell). Statistical significance, ****P > 0.0001. Ordinary one-way ANOVA Tukey multiple comparison test. Each data point represents the affinity for one T cell to a given monomer. Data represents 3 individual experiments.

Previous surface plasmon resonance (SPR) 3D affinity data has shown the APL 41M and 41C to have similar 3D affinities, with K_D_ values of 17 μM and 45 μM (50), 9.1 μM, and 12.2 μM (51) and 2.3 μM and 3.5 μM respectively (52). More recently, the advantages of 2D based affinity measurements over 3D SPR analyses have been highlighted using FRET (53) and the mechanical 2D-MP assay (4, 6, 22). The embedment of the TCR and pMHC within their respective cell membranes and the use of live cells in such 2D based analyses give a physiologically relevant portrayal of the TCR's native environment and the 2D restricted interaction between this receptor and its ligand. Furthermore, 2D affinity correlates closely with the potency of CD4 and CD8 T cells (6, 22, 41, 44, 53–55) making 2D-MP a key biophysical parameter. In our experiments, we used the 2D-MP and measured the 2D TCR affinity of LCMV specific naïve TCR-transgenic (P14) CD8 T cells to the three immunodominant GP33 epitopes presented by a mutant D^b^ monomer (D227K mutation of the MHC I α3 domain that abolishes CD8 binding) (6, 56–59). We found that 41M had the highest 2D affinity as compared to wildtype 41C and 41CGI, with mean population affinities of 1.04E-03 μm^4^, 6.62E-04 μm^4^, and 1.55E-04 μm^4^ respectively (Figures 1A). Furthermore, 2D-MP allowed us to take a single T cell of interest and probe against the three pMHCs. The data (Figure 1B) demonstrate the hierarchy of the three GP33 variants occurs at the level of each individual T cell. As a control, the order in which a given pMHC monomer was tested against the same T cell was randomized. These differences in 2D affinity are independent of any inherent changes in peptide affinity for MHC. Interestingly, while all three pMHC complexes had relatively high 2D affinities (as compared to 2D measurements of OT-I with cognate pMHC) (55), 41M had a ~2-fold higher affinity than 41C and ~9-fold higher affinity than 41CGI.

### CD8 contribution to TCR:pMHC binding does not alter the 2D TCR affinity hierarchy to 41M, 41C, and 41CGI.

**Figure 2:**
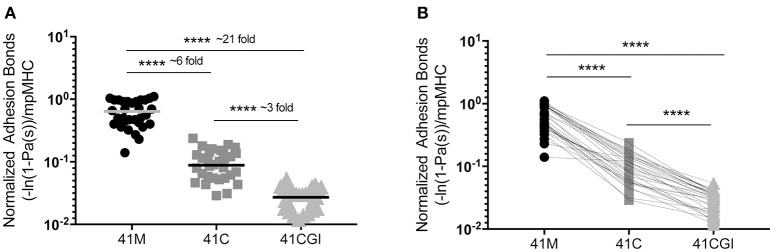
The contribution of CD8 binding to TCR:pMHC does not alter the hierarchy of bond formation between dominant GP33 epitopes. Normalized adhesion bonds for the trimolecular interaction of TCR:pMHC:CD8. **(A)** Shows the overall normalized adhesion bond mean ± SEM of cells tested to 41M, 41C or 41CGI. **(B)** Shows the individual normalized adhesion bonds of each P14 CD8+ T cell tested to all three pMHC monomers in **(A)**. Statistical significance, ****P > 0.0001. Ordinary one-way ANOVA Tukey multiple comparison test. Each data point represents the normalized bonds for one T cell to a given monomer. Data represents 3 individual experiments.

We next wanted to investigate the contribution of the CD8 co-receptor to the interaction between TCR and the pMHC complex. The affinity of CD8 is significantly lower than the affinity of TCR for pMHC (D^b^) (5, 46) but it is thought to enhance binding to ligands by the lowest affinity TCRs or aid recognition in the presence of low dose antigen (40). One can use wild type pMHC monomers, (intact D^b^-CD8 binding) to quantify the number of TCR:pMHC and CD8 to MHC bonds (41). Similar to our TCR:pMHC 2D affinity data, normalized adhesion bonds showed the same trend with 41M having a bond number higher than 41C which was higher still than 41CGI (mean bond numbers of 6.40E-01, 1.01E-01, and 2.93E-02 μm^2^ respectively) (Figure 2A). Using the same analysis of 2D-MP as in Figure 1B, a single T cell was probed against the three viral variants and the highest to lowest total bond numbers were determined as: 41M> 41C> 41CGI (Figure 2B). Additionally, these data demonstrated that the CD8 co-receptor contribution for these relatively high affinity pMHCs was different across the three epitopes (Figure 2A). These data highlighted CD8's ability to contribute differently with each variant epitope, further revealing the differences between 41M, 41C, and 41CGI.

### CD8 coreceptor binding bolsters bond lifetime under force but fails to restore the 11-Mer 41CGI to a bond lifetime comparable to either 41C or 41M

Previously, using optical tweezer technology, it has been demonstrated that T cells generate piconewton (pN) force in response to agonist pMHC (60, 61). The BFP assay can also be used to apply force to single TCR:pMHC bonds. Under force, the TCR:pMHC interaction can be divided into two types of bonds: bonds that strengthen the interaction between TCR:pMHC as force is applied (a catch bond) or TCR:pMHC interactions that generate a bond that weakens as force increases (a slip bond) (9, 10, 61).

**Figure 3:**
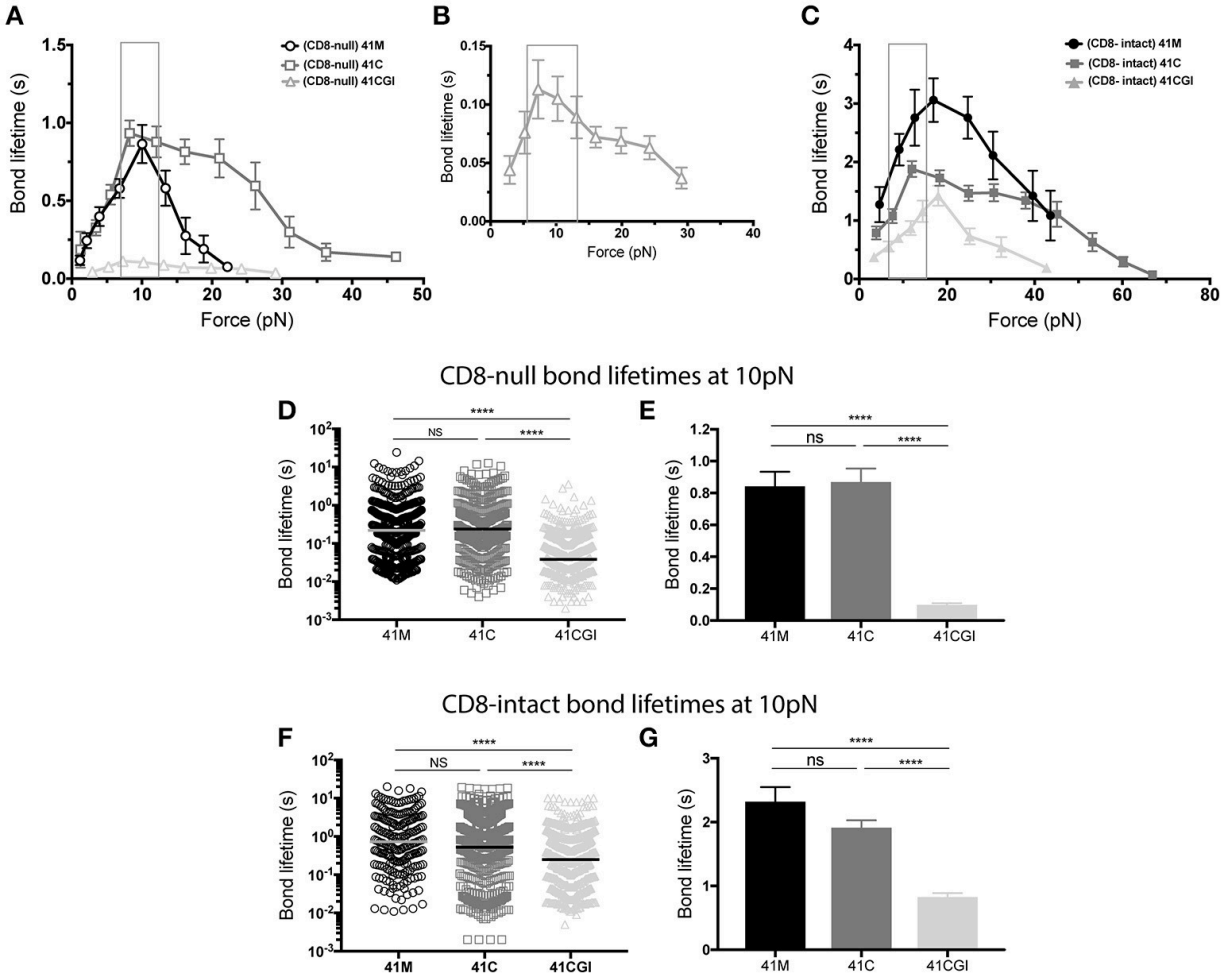
The 11-mer 41CGI has dramatically reduced bond lifetime under force in comparison to 41C and 41M even with the contribution of CD8 coreceptor. Bond lifetime under force **(A)** shows bond lifetime curves for 41M (open circle), 41C (open square) and 41CGI (open triangle) with CD8-null pMHC **(B)** shows bond lifetime curve for 41CGI with CD8-null pMHC with a smaller scale. **(C)** Shows bond lifetime curves for 41M (closed circle), 41C (closed square) and 41CGI (closed triangle) with CD8-intact pMHC. CD8-null bond lifetimes at ~10 pN of force for all three variants shown as **(D)** a scatter plot or **(E)** a bar graph. CD8-intact bond lifetimes at ~10 pN of force for all three variants shown as **(F)** a scatter plot or **(G)** a bar graph. All force curves generated >1,000 bond lifetimes.

Here, we apply force to the TCR:pMHC bond with and without the contribution of CD8 for all three pMHC variants (Figures 3A–C). In each case, the P14 TCR exhibits a catch bond, which is intensified by the contribution of CD8 (Figure 3C). The bond lifetimes under force also revealed a hierarchy amongst the peptide antigens. In the absence of CD8, 41M had a peak bond lifetime of ~0.8 s (Figure 3A), which was similar to 41C with a lifetime of ~0.9 s (Figure 3A), both of which were significantly higher than 41CGI which had the lowest bond lifetime of ~0.1 s (Figures 3A,B).

Liu et al. have revealed CD8 coreceptor to be critical for T cell mediated force generation and cell spreading (62). Therefore, we next wanted to investigate the previously uncharacterized CD8 coreceptor contribution to the TCR:pMHC complex under force. Thus, we generated force curves that measured the bond lifetime of TCR bound to CD8-intact pMHC monomers. Our data demonstrated that CD8 contribution significantly increased bond lifetime under force for all three peptide variants, with peak bond lifetimes showing a ~3-fold increasing from ~0.8 to ~3.0 s (Figures 3A,C) for 41M while 41C showed a ~2-fold increase from ~0.9 to ~1.9 s (Figures 3A,C). More interesting perhaps was the contribution of CD8 to TCR bond lifetimes under force with 41CGI, which exhibited a ~15-fold increase from ~0.1 to ~1.5 s (Figures 3B,C).

Using the BFP assay, we and others have shown that the bond lifetime of a given TCR:pMHC pair changes when the applied force is varied (6, 9–11). Interestingly, we (10, 63) and others (61, 64) have also shown that peak bond lifetime is often observed at ~10pN for both CD4 and CD8 T cells (61). Additionally, Liu et al. have revealed that naïve T cells can naturally exert 12-19pN of force on their TCRs within seconds of ligation (62). Therefore, we highlighted ~10pN as the physiologically relevant point of comparison of the force curves generated with the three GP33 variant monomers. As such, we found that at 10pN of force, 41C has a longer bond lifetime than 41M and dramatically more so than 41CGI (Figures 3D,E). However, in the presence of CD8, there is no significant difference between 41M and 41C (Figures 3F,G). These data highlight the dramatic contribution of CD8 coreceptor toward the weakest variant 41CGI.

### 2D affinity and bond lifetime under force are indicative of early T cell triggering and T cell function

**Figure 4:**
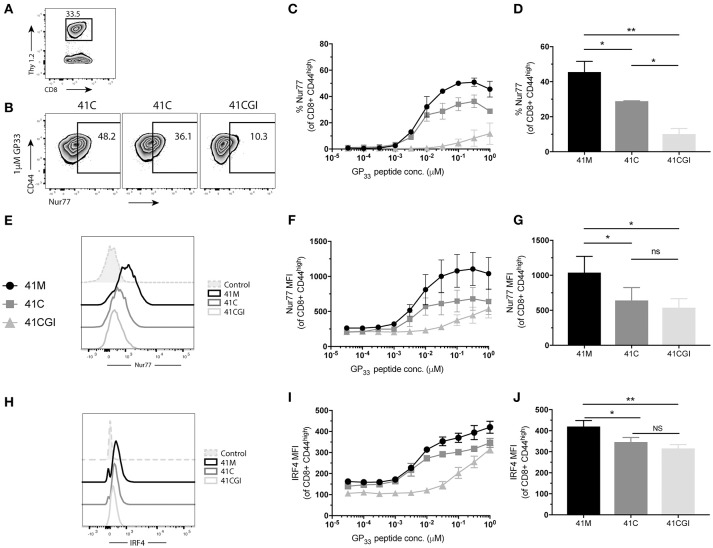
41M stimulated P14 CD8+ T cells perceive antigen more strongly than either 41C or 41CGI *ex vivo*. Naïve Thy1.2 P14 CD8+ T cells (1 × 10^4^) were transferred into Thy1.1 congenic B6 hosts that were infected with LCMV Armstrong a day later. Splenocytes were harvested at D8 p.i. and re-stimulated for 1 h *ex vivo* with the three peptides at a range of doses **(A)** Representative contour plot for P14+ Thy1.2 CD8 T cell gating strategy. **(B)** Representative flow cytometry plot gating Nurr77 staining (pre-gated on P14s (CD8+Thy1.2) with 1 uM peptide - left 41M, center 41C and right 41CGI. **(C)** Shows %Nur77+ at a range of doses. **(D)** Representative Nur77+ staining at 1 uM for each peptide. **(E)** Nur77 MFI at a range of doses. **(F)** A representative histogram of Nur77 MFI staining using 1 uM peptide. **(G)** Shows a representative experiment with Nur77 MFI staining at 1 uM **(H)** IRF4 MFI at a range of doses. **(I)** A representative histogram of IRF4 staining using 1 uM peptide. **(J)** Shows a representative experiment with IRF4 MFI staining at 1 uM. Data shown are representative of 3 experiments. *n* = 3-4 mice per experiment. Bar graphs with mean ± SEM. Statistical significance, ns = no significance, *P > 0.05, **P > 0.01. Ordinary one-way ANOVA Tukey multiple comparison test.

Next, we determined the effect of the 2D kinetics on the T cell response. To assess whether TCR signal from either 41M, 41C or 41CGI was perceived similarly, transgenic CD8+ P14 T cells were adoptively transferred into congenic hosts (Figure 4A) which were then infected with LCMV Armstrong a day later. Spleens were harvested at peak infection (D8) and cells were re-stimulated *ex vivo* with each variant peptide. The orphan nuclear hormone receptor, Nur77, has been shown to be rapidly upregulated in T cells stimulated with antigen via the TCR, but not by inflammatory stimuli (65), and has also been correlated with the strength of the TCR stimulus. Additionally, interferon regulatory factor-4 (IRF4) has been implicated in T cell differentiation and expansion and has been suggested to correlate with the stimulatory potency of a given pMHC (66–68). Our data demonstrated that a higher percentage of CD8+ T cells re-stimulated with 41M expressed Nur77 (Figures 4B,E) and have a higher MFI (peaking at 0.31 uM) than 41C and dramatically more so than 41CGI (Figures 4C,D,F,G). Similarly, 41C stimulated cells had significantly higher frequencies of Nur77+ CD8+ T cells than those stimulated with 41CGI (Figures 4C,D). However, while there was a difference in IRF4 MFI (Figure 4H) between 41M, 41C, and 41CGI when cells were stimulated at low peptide doses, we did not observe a difference between 41C and 41CGI at the highest peptide dose tested of 1 uM (Figures 4I,J). These data, while not factoring in peptide loading, indicated that at equivalent peptide doses, ligand potency can correlate with TCR:pMHC 2D affinity (41M > 41C > 41CGI).

**Figure 5:**
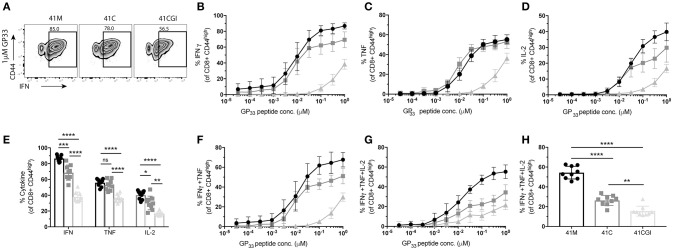
P14 cytokine production correlate with 2D affinity for the three variants. Naïve Thy1.2 P14 CD8+ T cells (1 × 10^4^) were transferred into Thy1.1 congenic B6 hosts that were infected with LCMV Armstrong a day later. Splenocytes were harvested at D8 p.i. and re-stimulated *ex vivo* for 6 h with the three peptides at a range of doses. **(A)** Representative contour plot for %IFNγ (pre-gated on CD8+) left 41M, center 41C and right 41CGI peptide. **(B)** Shows IFNγ, **(C)** TNFα and **(D)** IL-2 cytokine production for a range of peptide doses. **(E)** Shows individual cytokine production at 1uM peptide. Dose response curves for **(F)** double production of IFNγ and TNFα, **(G)** triple production of IFNγ, TNFα and IL-2 and **(H)** shows the triple producers at 1uM peptide concentration. Data shown are representative of 3 experiments *n* = 3-4 mice per experiment. Bar graphs with mean ± SEM. Statistical significance, ns = no significance, *P > 0.05, **P > 0.01, ***P > 0.001, ****P > 0.0001. Ordinary one-way ANOVA Tukey multiple comparison test.

Where TCR signal transduction is detectable seconds after TCR engagement with pMHC, cytokine production is observed within hours. Using the same experimental design, we examined cytokine production for the three variants. We show that in response to 41M (which displayed the highest 2D affinity and perceived signal strength) cells also produced the most IFNγ (Figures 5A,B) and IL-2 (Figure 5D) than either 41C or 41CGI stimulated P14s. However, 41M and 41C activated cells produced similar amounts of TNF (Figures 5C,E). A significantly higher frequency of P14s were also double producers of IFNγ and TNF (Figure 5F) and triple producers of IFNγ, TNF and IL-2 (Figure 5G) with 41M stimulation than post activation with either the 41C or 41CGI peptides (Figure 5H).

**Figure 6:**
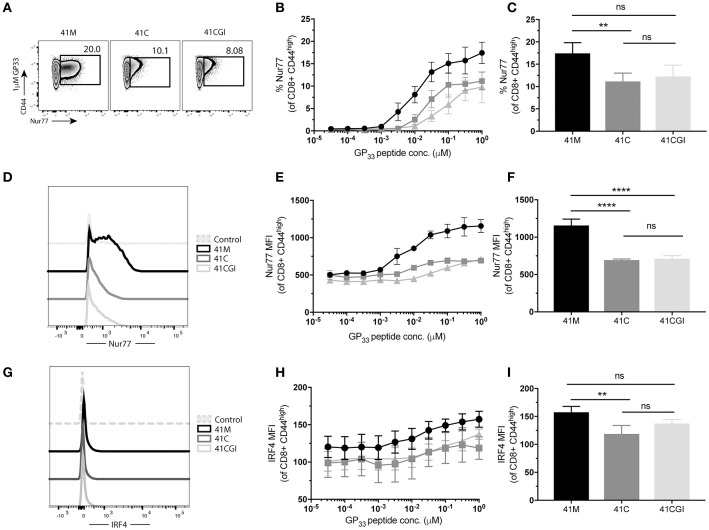
Polyclonal CD8+ T cells recognize 41M more intensely than 41C or 41CGI. B6 mice were infected with LCMV Armstrong and splenocytes harvested at D8 p.i. and re-stimulated with either 41M, 41C or 41CGI *ex vivo* **(A)** Representative contour plot for nur77 (pre-gated on CD8+) for left 41M, center 41C, and right 41CGI peptide stimulations. **(B)** Shows %Nur77+ at a range of doses. **(C)** Representative %Nur77 staining at 1uM for each peptide. **(D)** Representative histogram of Nur77 MFI staining at 1 uM for each peptide. **(E)** Shows Nur77+ MFI at a range of doses. **(F)** Representative Nur77+ MFI staining at 1 uM for each peptide. **(G)** A representative histogram of IRF4 staining using 1 uM peptide **(H)** IRF4+ MFI at a range of doses. **(I)** Shows a representative experiment with IRF4 MFI staining at 1uM.Data shown are representative of 3 experiments *n* = 3-4 mice per experiment. Bar graphs with mean ± SEM. Statistical significance, ns = no significance, **P > 0.01. Ordinary one-way ANOVA Tukey multiple comparison test.

We next wanted to investigate the impact of these peptide variants on the functional response of polyclonal CD8+ T cells. Thus, we infected C56BL/6 mice with LCMV Armstrong and splenocytes were harvested at peak infection then re-stimulated *ex vivo* with our peptide variants. Similar to our observation with monoclonal P14 cells, more polyclonal CD8 T cells exhibited increased frequencies of Nur77 expression upon stimulation with 41M as compared to 41C and 41CGI (Figures 6A–D) but Nur77 MFI for 41C and 41CGI was not significantly different (Figure 6F). However, IRF4 expression revealed only a slight difference between 41M and 41C at the highest peptide dose of 1 uM (Figures 6E–I).

## Discussion

The ability of an antigen specific TCR to be triggered and sufficiently induce activation related changes in the T cell that lead to clonal expansion and cytokine production is primarily dictated by early interactions with antigen. Identifying how TCR triggering equates to functional outcome and fate is still being investigated. Much of our understanding of TCR affinity for pMHC and functional outcomes stems from the use of APLs. For the most part, APLs are assumed to be of lower affinity for the TCR based on reduced functional responses. For many APLs exemplified by self and antagonist epitopes, SPR measurements lacked sensitivity to measure binding kinetics. The OT-I OVA system and its ligands that display high to low functional responses have been used to dissect their direct influence on CD8 T cell effector functions and memory responses (55, 69–71). The 3D affinity for many of the OT-I APLs (71) has not been reported although we have analyzed several for 2D affinity (6, 55). Here, we build on the extensive knowledge of the GP33 immunodominant epitopes, evaluating the correlation between 2D kinetics of affinity and bond lifetime using three variant agonist antigens.

GP33 is documented as being one of the three immunodominant CD8 H2-D^b^ restricted epitopes for LCMV in C57BL/6 mice, along with GP_276−286_ and NP_396−404_. Numerous studies have highlighted the contributing factors that confer immunodominance for a given peptide, namely stability of the peptide:MHC I complex, antigen processing and presentation, and TCR:pMHC I binding affinity (72–74). To this background we now overlay the contribution of 2D affinity and bond lifetime under force. Our data add insight and highlight the importance and striking contrast between the most proximal step: TCR 2D affinity for pMHC and the ensuing TCR bond lifetime for pMHC under force. Furthermore, we emphasize the capability of 2D-MP to probe single receptor:ligand interactions and resolve subtle but significant changes in peptide binding that are independent of peptide loading.

Gairin et al. demonstrated, based on binding, that the 9-mer (41C) and the 11-mer (41CGI) are the immunodominant GP33 epitopes. In RMA-S MHC stability assays (D^b^ K^b^), the 9-mer 41C was six times more efficient than 41CGI at inducing upregulation of H-2D^b^. However, in competition experiments performed on the T-2D^b^ cell line, the 11-mer 41CGI was six times more efficient at competing for pMHC than the 9-mer 41C (8). The question is to what extent loading of MHC affects the T cell response and their biology. For example, 41M and 41C epitopes in our experiments had the same sensitivity to antigen, which would argue similar effective loading. In addition, others have reported biological differences in T cell responses and migration (interaction with APCs) that 3D affinity would not explain (51, 52). In the lymph node (LN), T cell motility has been labeled as having two distinct phases of behavior (36). Phase one type behavior consists of rapid T cell movements with multiple short sampling encounters with dendritic cells (DCs) before progression to a second phase comprised of stable and long lasting contacts with DCs (75). Henrickson et al. showed T cells interacting with 41M pulsed DCs transitioned more quickly to phase two than upon encountering 41C. We would suggest that the differences in 2D kinetics are integral to the different sampling phenotypes with 41C and 41M. In particular, one could envisage force being important to the biological migration observations to LFA-1 (76).

Several studies have reported the SPR derived 3D affinities for wildtype GP33 (41C) and the 41M mutation with ranges from 2 to 45uM (50–52). At these levels, there is probably no difference in P14 TCR affinity for 41C or 41M. For example, Boulter et al. show that 41C and 41CGI have very similar 3D affinities of 2.3 and 3.5 uM, respectively. The 2D measurements displayed at least a 2-fold difference. We have previously identified 2-fold or greater differences in affinity can have profound changes in function (6, 55, 77–79), which would be consistent with the 41C giving larger responses as read out by Nur77, IFNγ and IL-2. Of note, one cannot directly compare 2D and 3D affinities as 3D affinities are measured in molarity and 2D affinities are measured in area. Instead, the bond lifetimes could provide a point of comparison. By SPR, 41C and 41M show bond lifetimes of ~0.5s (51) which is similar to what we find by BFP (0.8–0.9s) for the TCR alone. A major difference in overall force occurs when the effect of CD8 is included as it increases the number of total bonds and the overall catch bond properties. These differences, which likely affect T cell biology, would not be apparent from the SPR assessment of P14 TCR for the respective GP33 peptides.

The crystal structures of several of the GP33 pMHC complexes have been reported with no major differences that explain our 2D kinetic findings (35). In general analysis of TCR:pMHC, crystal structures have failed to identify obvious factors for catch bond. TCR interactions that cause force are dynamic and occur optimally with CD8, Lck and the cell cytoskeleton (62). In recent collaborative work, it was found that the static crystal structures of pMHC with TCR alone did not identify the type of bond that will be formed under force. However, molecular dynamic simulations (MSD) could be used to add pulling force on the molecules and begin to identify key features (9).

While 2D kinetics are not impacted by peptide loading as they are single molecule interactions, it is important to note that functional differences might be impacted by peptide loading. However Gairin et al. show that, based on binding, different assays give varying results on whether 41C or 41CGI is most stable (8). Our 2D kinetics clearly outline the hierarchy of GP33 epitopes and these data correlate with early signaling events. Using P14 adoptive transfer into congenic hosts studies wherein mice were injected with LCMV Armstrong and then re-stimulated *ex vivo* with our three peptides, we demonstrated that 41M stimulated P14 CD8 T cells give a stronger signal than 41C as measured by Nur77 upregulation. Additionally, both 41M and 41C stimulated cells induce significantly stronger signals than 41CGI stimulated cells. While Nur77 is often used as a readout of signal strength and indicative of functionality (80, 81), the type of CD8 function cannot clearly be deduced without further analysis. Using the same experimental design, we observed significantly diminished cytokine production in 41CGI stimulated P14 CD8 T cells as compared to 41M and 41C, and the same hierarchy for P14 CD8 T cells producing IFNγ and IL-2 although 41M and 41C result in similar amounts of TNFα at the concentrations used here. Here we show 2D affinity to correlate with triple cytokine production in P14 transgenics. These findings are replicated using polyclonal CD8 T cells taken from D8 LCMV Armstrong infected mice re-stimulated with the different peptides. The 2D affinity distribution for a P14 monoclonal population spans a ~10-fold range while the GP33 specific polyclonal CD8 T cell population can encompasses a wider ~1,000-fold affinity range (82, 83) but nevertheless the frequency hierarch of triple cytokine producers in response to the three variants is preserved in both despite differences between monoclonal and polyclonal populations and any differences in peptide loading. Moreover, we have demonstrated that using 41M and its viral escape mutant 35A that while peptide loading is lower with 35A, in a polyclonal LCMV Armstrong infection 2D affinity is not significantly different (84).

Although T cell responses are aggregations of the TCR bond lifetimes, bond lifetime under force is a single molecule measurement independent of peptide loading and is a critical parameter in determining T cell functionality (6, 10, 22). While 41CGI had the lowest 2D affinity of the three, it still had a relatively high 2D affinity. Given its high affinity, its low peak bond lifetime of ~0.1 s was somewhat surprising (Figures 3A,B). We have similarly recently analyzed the HLA B35-HIV epitope and found a high affinity TCR possessing a short bond lifetime that, in this case, showed slip bond characteristics. Together, this demonstrated that affinity and bond lifetime are not always directly correlated (9). Our data show that at 10pN of force, with or without CD8 contribution there is no significant difference in bond lifetime between 41M and 41C suggesting that any difference in functional responses between 41M and 41C based from assays in this study could be due to differences in 2D affinity.

Unlike the OT-I APL system where often the question is how 2D affinity differences elicit changes in the generation of effector and memory T cell populations, here the question is how the use of GP33 variants might change the perception of the GP33 epitope immunodominance within the CD8 response to LCMV. Our data clearly show that 41M elicits higher 2D affinity, increased number of total bonds with CD8 and longer bond lifetime under force as well as a more robust CD8 T cell response than both 41C and 41CGI. Super agonists are ligands for the TCR that stimulate the T cells more than the processed antigenic peptide (85). By our 2D affinity measures and functionality, 41M would be defined as a super agonist peptide variant as it possesses a prominent difference between 41CGI and the truncated 41C. This raises the question of whether using the super agonist 41M in lieu of 41C or 41CGI gives an accurate interpretation of the efficacy of the GP33 targeted response relative to the other immunodominant CD8 LCMV epitopes (GP276 and NP396). Furthermore, our data highlight how the CD8 co-receptor engagement with TCR:pMHC can change with viral variants providing another point to consider. Lastly, our work demonstrates the power and sensitivity of 2D kinetic measurements in demonstrating how TCRs can determine subtle differences in related agonist ligands that can potentially lead to different functional outcomes.

## Author contributions

EK and BE conceived the project and wrote the manuscript. EK and BL performed BFP experiments. EK and RA performed 2D-MP experiments. EK and JJ performed intracellular flow cytometry experiments. EK analyzed all data. BL and RA discussed findings and RA edited the manuscript.

### Conflict of interest statement

The authors declare that the research was conducted in the absence of any commercial or financial relationships that could be construed as a potential conflict of interest.
